# Gelatine Backing Affects the Performance of Single-Layer Ballistic-Resistant Materials Against Blast Fragments

**DOI:** 10.3389/fbioe.2020.00744

**Published:** 2020-07-02

**Authors:** Thuy-Tien N. Nguyen, George Meek, John Breeze, Spyros D. Masouros

**Affiliations:** ^1^Department of Bioengineering, Imperial College London, London, United Kingdom; ^2^Royal Centre for Defence Medicine, Queen Elizabeth Hospital Birmingham, Birmingham, United Kingdom

**Keywords:** ballistic fabrics, personal protective equipment, body armor, blast injury, testing standard, injury, protection

## Abstract

Penetrating trauma by energized fragments is the most common injury from explosive devices, the main threat in the contemporary battlefield. Such devices produce projectiles dependent upon their design, including preformed fragments, casings, glass, or stones; these are subsequently energized to high velocities and cause serious injuries to the body. Current body armor focuses on the essential coverage, which is mainly the thoracic and abdominal area, and can be heavy and cumbersome. In addition, there may be coverage gaps that can benefit from the additional protection provided by one or more layers of lightweight ballistic fabrics. This study assessed the performance of single layers of commercially available ballistic protective fabrics such as Kevlar^®^, Twaron^®^, and Dyneema^®^, in both woven and knitted configurations. Experiments were carried out using a custom-built gas-gun system, with a 0.78-g cylindrical steel fragment simulating projectile (FSP) as the impactor, and ballistic gelatine as the backing material. FSP velocity at 50% risk of material perforation, gelatine penetration, and high-risk wounding to soft tissue, as well as the depth of penetration (DoP) against impact velocity and the normalized energy absorption were used as metrics to rank the performance of the materials tested. Additional tests were performed to investigate the effect of not including a soft-tissue simulant backing material on the performance of the fabrics. The results show that a thin layer of ballistic material may offer meaningful protection against the penetration of this FSP. Additionally, it is essential to ensure a biofidelic boundary condition as the protective efficacy of fabrics was markedly altered by a gelatine backing.

## Introduction

Penetrating trauma due to energized fragments is the most common cause of injury from an explosive event (Bowyer, 1996; Hill et al., 2001; Covey, 2002; Champion et al., 2009; Eastridge et al., 2012). Fragments can be part of explosive devices (primary fragments) such as shrapnel from munition casings and objects purposely included in home-made explosive devices such as nuts, bolts, and ball bearings (Hull, 1992; Hill et al., 2001; Abbotts et al., 2007; Weil et al., 2007; Brunner et al., 2015). They can also be part of the environment in the vicinity of the explosion (incidental, secondary fragments) such as glass shards and debris from collapsed structures, or even foreign bone fragments from other victims (Hull, 1992; Abbotts et al., 2007; Brunner et al., 2015; Dick et al., 2018; Kalem and Ercan, 2018). They are propelled and accelerated by the energy of the explosion to an initial velocity of the order of 1000 m/s, quickly decelerate. One analysis of casualties survived for assessment in a medical treatment facility suggested that they were likely to have been impacted by fragments of less than 600 m/s (Bowyer, 1996). Fragment penetrations can result in lethal hemorrhage in the truncal region (Eastridge et al., 2012; Breeze et al., 2016); the most commonly affected body regions have been shown to be the extremities and the head (Owens et al., 2008; Breeze et al., 2015; Penn-Barwell et al., 2015). Owens et al. (2008) reported that of 5,155 combat wounds by an explosive mechanism (78% of all injuries) in the Operation Iraqi Freedom and Operation Enduring Freedom, 44% were to the extremities and 26% were to the head and neck area. Penn-Barwell et al. (2015) found that 70% of 2,792 United Kingdom combat casualties in Iraq and Afghanistan between 2003 and 2012 were caused by explosions with 43% to the extremities and 24% to the head and neck region. Breeze et al. (2015) also showed that lower extremities and face are the most penetrated regions by blast fragments; they also demonstrated that the use of personal armor can reduce effectively the number of injuries.

Body armor has been long used to protect against ballistic threats and is generally categorized as hard armor and soft armor (Breeze et al., 2016). Hard armor often consists of composite or ceramic plates and is mainly used to protect the thorax and abdomen (Breeze et al., 2016; Neves Monteiro et al., 2017). It can achieve high levels of protection, but can be heavy and cumbersome (Tilsley et al., 2018). Soft armor typically consists of flexible fabric panels, commonly in knitted or woven form, made from synthetic aromatic polyamides (aramids) or high-performance polyethylene (HPPE). In the woven fabric, the material fibers mesh and overlap each other; the plain weave where the warp and weft fibers are orientated perpendicularly and overlapped alternatively is the most common woven type (Tran et al., 2014). In knitted fabrics, the loops of the material fibers are linked together to form the structure (Tran et al., 2014). Another type of armor textile is the felt fabric where the material fibers are needle-punched in random orientations to entangle together into the resultant product (Chocron et al., 2008). Soft body armor cannot currently provide the same level of protection as hard body armor, but with the advancement of currently available ballistic fabrics, it provides protection against energized fragments (Breeze et al., 2016). Soft armor used in a military body armor vest varies in its construction, but comprises 10–50 layers of fabric and may weigh up to 9 kg (Sakaguchi et al., 2012); conversely, it has been shown that one or two layers of fabric may provide meaningful protection while not adding significant weight and stiffness, and still allow for heat dissipation (Carr, 1999; Sakaguchi et al., 2012). The United Kingdom Armed Forces are supplied with Tier 1 pelvic protection which consist of two knitted layers made from an elastic silk material; this has been shown to reduce the incidences of surface pelvic injury by 48% (Breeze et al., 2015). There was also evidence from the initial fielding in Afghanistan that this PPE could reduce the ingress of debris, hence reducing damage to the upper thigh and perineal area (Lewis et al., 2013). The study by Sakaguchi et al. (2012) suggests that one or two layers of a woven aramid material can offer some protection to the extremities against low energy fragments, and that two layers of the same fabric only offer 10–25% of improvement in perforation thresholds compared to one layer. Breeze et al. (2013a) also recommended the use of thin layers of ballistic material to protect the neck, which was subsequently incorporated into the under body armor vest.

An important aspect in the application of body amours is the ballistic testing of their protective performance. The two most often used standards for this assessment are the NATO AEP-2920 procedures for the evaluation and classification of personal armor (NATO, 2016) and the ballistic resistance of body armor NIJ Standard-0101.06 (NIJ, 2008). The backing material is not always recommended for soft armor; in the NATO AEP-2920 procedure, a back-face signature material is only mandatory for evaluating V_proof_ (the velocity at which the probability of the armor being perforated by the projectile is less than 10%, with confidence interval level of 90%), and not for V_50_ (the velocity at which the probability of the armor being perforated by the projectile is less than 50%). When applicable, the recommended backing material by these standards is plasticine; this material, however, has quite different properties compared to various tissues of the human body (Genov, 2012).

The aim of this study is (i) to assess and compare the protective behavior of a range of commercially available ballistic fabrics, in single-layer construction with ballistic gelatine backing material, against a small metallic blast fragment; and (ii) to investigate whether this behavior depends or not on the presence of the backing material. An additional aim was to investigate various criteria for evaluating the ballistic performance as a basis for recommendation to future testing.

## Materials and Methods

**FIGURE 1:**
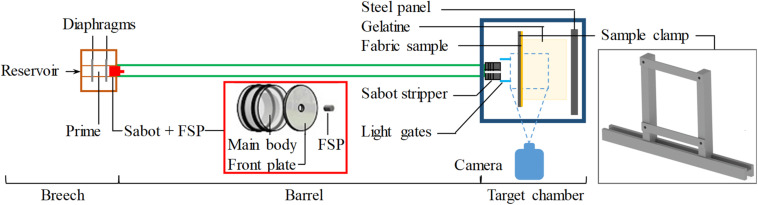
Schematic of the gas gun experimental set-up.

Impacts by fragment simulating projectile (FSP) on ballistic materials were carried out using a 32-mm-bore single-stage gas-gun system (Figure 1) as described by Nguyen et al. (2020). The primary choice of FSP for this study was the 0.78-g carbon steel cylinder based on the study by Breeze et al. (2013d), which was designed to be 4.5 mm in diameter so that it is in accordance with the NATO standardized agreement of a ballistic test method for personal armours (NATO, 2016). High-speed photography (AMETEK Vision Research VEO710L, United States) was used to record the event and to estimate the impact velocity of the FSP on the tested material.

Seven ballistic materials (Table 1) chosen for testing were Kevlar^®^ and Twaron^®^ aramids which are the two most well-documented commercial ballistic fibers, Dyneema^®^ HPPE, and a technology of highly strain-rate sensitive polymer with a range of functional geometries laminated with woven Kevlar^®^ – from here on is referred to as “dilatant fabric.”

**TABLE 1:** Ballistic materials chosen for assessment with their areal density and mounting condition.

**Material**	**Areal density [g/m^2^]**	**Mounting condition**
Kevlar^®^ plain weave	125	400 × 400 mm panel with all sides rolled up into 100 × 100 mm test area
Twaron^®^ plain weave 1	125	
Twaron^®^ plain weave 2	190	
Kevlar^®^ felt	275	
Dilatant fabric	300	
Kevlar^®^ knit	260	Clamped at all sides to 100 × 100 mm test area
Dyneema^®^ HPPE knit	610	

These materials were tested in a single-layer configuration. The samples were mounted as described in Table 1 to achieve a suitable boundary condition recommended by the manufacturers. All fabric samples were clamped taut with no specific stretch to be as similar as possible to the condition during usage. It was ensured that no more than three shots were performed on each 100-by-100-mm test area of each sample, and that the impacts were spaced evenly over the test area.

Twenty percent by weight ballistic gelatine (type A, 300 bloom) was used as the backing material in the impact tests (Breeze et al., 2013b; Nguyen et al., 2018). The ballistic gelatine is a widely used subdermal soft-tissue simulant as it has been shown to have similar response to human tissue (Shepherd et al., 2009; Appleby-Thomas et al., 2011; Breeze et al., 2013c, b; Kieser et al., 2014). Especially, Breeze et al. (2013b) reported that the 20% gelatine simulant has comparable behavior to the leg and neck muscles of porcine specimens against penetration by metallic FSPs. In addition, gelatine has similar deformation response to Roma Plastilina No. 1 in terms of behind soft armor blunt trauma assessment (Prather et al., 1977; Hanlon and Gillich, 2012). A 250 × 150 × 50 mm gelatine block was put lightly in contact with, and behind, the sample to simulate a realistic boundary condition between the ballistic fabric and the soft tissue. Post impact, the tested material was inspected for visual signs of deformation. The resulting depth of penetration (DoP) in the tissue simulant was measured with a ruler after cutting the gelatine transversely to the travel path of the FSP.

The performance of the sample was assessed across five categories:

(a) Impact velocity of FSP at 50% probability (V_50_) of perforation through the fabric, i.e., the FSP broke the material and escaped from the back of it.
(b) V_50_ of penetration into the ballistic gelatine regardless of depth of penetration.
(c) V_50_ of penetration into the ballistic gelatine with a depth of more than 15 mm, as an indicator of a high risk of injury. This DoP was chosen based on a range of minimum depths from the skin surface to essential structures: these were reported as 17 mm to the liver, 19 mm to the heart, and 15 mm to the common carotid artery in the lower third of the neck in the United Kingdom military population (Breeze et al., 2013a, 2020); an article on a non-military population using ultrasound demonstrated this depth being 14.6 ± 5.1 mm to the femoral artery (Seyahi et al., 2005) (to our knowledge, no figure has been published for a representative military population).
(d) Depth of penetration in gelatine for each impact velocity tested.
(e) The normalized percentage energy absorption of the material calculated as:

$$\hat{E}=\left(\frac{v_{0}^{2}-v_{1}^{2}}{v_{0}^{2}}\right)/\rho_{A},$$

where for the same DoP in gelatine, *v*_*0*_ and *v*_*1*_ are, respectively, the impact velocity of the FSP with and without the ballistic material, and ρ_*A*_ is the areal density of the material. This variable indicates the efficiency of the material in terms of protective performance per unit of mass.

To obtain the three values of V_50_ in categories (a)–(c), a risk curve was generated for each category using Weibull survival analysis (Quenneville et al., 2011; Yoganandan et al., 2016) conducted with the NCSS statistical software (Utah, United States). The Weibull regression model was used with impact velocity of the FSP as the predictor variable; tests were classified as left censored if resulting in perforation/penetration and as right censored otherwise. For data sets with distinct gap in the impact velocities between injurious and non-injurious cases, a step function was fitted to obtain the V_50_ values.

To investigate the effect of the soft-tissue simulant on the injurious outcome, the same experimental model was repeated, but without the ballistic gelatine backing, on five ballistic materials: Dyneema^®^ HPPE knit, Kevlar^®^ plain weave, Twaron^®^ plain weave 2, Kevlar^®^ felt, and Kevlar^®^ knit. Only the first category, V_50_ of perforation of tested material by the FSP, was used to quantify the performance of the fabrics tested in this boundary condition. The V_50_ values were obtained using two different methods: (i) the Weibull survivability analysis as described earlier; and (ii) the arithmetic mean evaluation described by the AEP-2920 NATO Standard (NATO, 2016) where it is calculated by averaging the three highest impact velocities that resulted in no material perforation and the three lowest impact velocities that resulted in material perforation (with the difference between the lowest and highest impact velocities less than 60 m/s, ideally less than 40 m/s). Values of V_50_ by these two methods were compared to assess their suitability, and the Weibull V_50_ values were compared with those of the set-up with gelatine backing to determine the relevance of including the soft-tissue simulant in the testing methodology.

## Results

Seventy-nine tests were performed in the set-up using ballistic gelatine backing with an average of eleven shots per fabric and at least nine shots for each fabric. For the boundary condition without soft-tissue simulant, fifty-four tests were carried out with an average of nine shots per fabric and at least six shots for each fabric.

### Performance of Commercial Ballistic Materials

**FIGURE 2:**
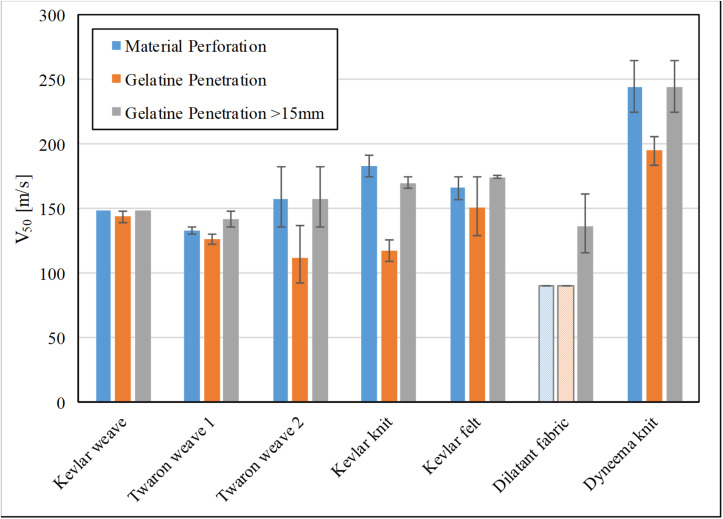
The FSP impact velocity with 50% risk of (a) material perforation (blue), (b) any penetration in gelatine (orange), and (c) >15 mm penetration in gelatine (gray) for each investigated ballistic fabric in single-layer format. Hatched bar indicates that V_50_ is less than this value as no further test below this impact velocity was conducted.

The FSP impact velocities giving 50% risk of material perforation, gelatine perforation of any depth, and gelatine perforation of more than 15 mm depth for each tested fabric are shown in Figure 2. For V_50_ values obtained from the Weibull survivability analysis, error bars show the 95% confidence interval of the fitting solution. For V_50_ values obtained from the step function fitting, error bars are the half-width value of the gap in the corresponding data set. For the dilatant fabric, material perforation and any gelatine penetration occurred in all tests whose FSP impact velocities were between 92 and 258 m/s. Hence, its V_50_ values for these categories were assumed to be less than 92 m/s and shown as hatched bars in Figure 2.

**FIGURE 3:**
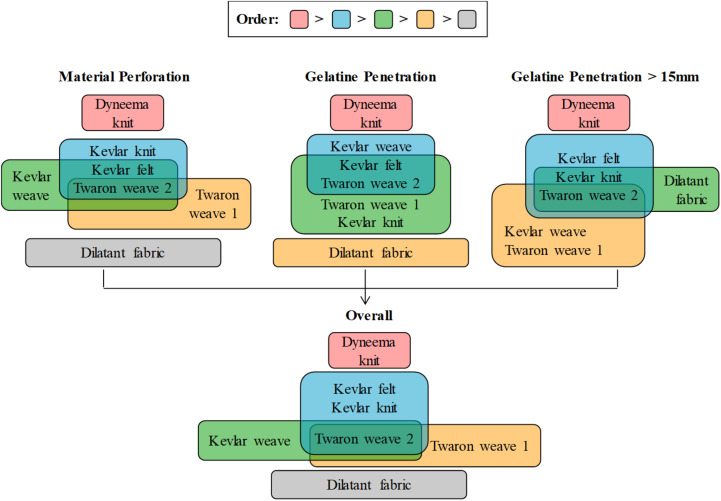
Groupings of tested fabrics with statistical *z*-test and Bonferroni multiple-comparison correction, α_*corrected*_ = 0.05/21≈0.002. Ballistic fabrics that do not belong to the same group showed significant difference in their V_50_ values. The color of the group reflects its order; there is no order for materials within the same group.

The statistical *z*-test with the Bonferroni multiple-comparison correction (α_*corrected*_ = 0.05/21≈0.002) was performed on the V_50_ values to group the materials in the corresponding category (Supplementary Tables SA–SC). For the statistical test, it was assumed that the survival analysis returned the V_50_ value equivalent to that as if obtained as a mean, with corresponding standard deviation, from a large number of tests following a normal distribution. To determine the overall groupings for all three categories, materials which were significantly different in at least two categories were deemed different from each other overall (Supplementary Table SD). Figure 3 exhibits the resultant groupings for performances of the investigated fabrics in terms of material perforation, gelatine penetration of any depth, gelatine penetration of more than 15 mm in depth, and overall behavior. The materials which did not belong to any common group showed significant difference (*p* < 0.002) in their behavior. There were no two materials with significant difference in only one category assuring that the overall group was reasonably reliable. In general, the ballistic fabrics were grouped into Group 1: Dyneema^®^ HPPE knit – the best performing material; followed by Group 2: Kevlar^®^ felt, Kevlar^®^ knit, Twaron^®^ weave 2; Group 3: Twaron^®^ weave 2, Kevlar^®^ weave; Group 4: Twaron^®^ weave 2, Twaron^®^ weave 1; and finally Group 5: the dilatant fabric.

**FIGURE 4:**
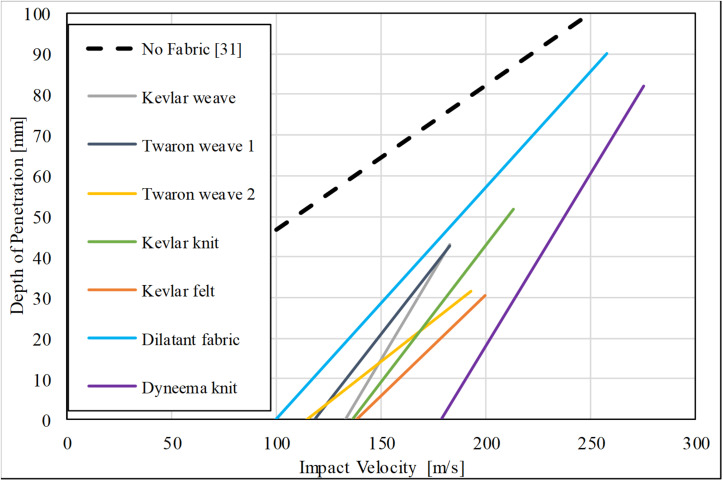
The DoP in gelatine backing material against FSP impact velocity for each investigated ballistic fabric in single-layer configuration [category (d)]. The data points are not displayed for clarity. The trend for gelatine only, i.e., no fabric, was obtained from Nguyen et al. (2018).

Figure 4 shows the DoP against FSP impact velocity for each ballistic fabric. The trend for each material was obtained for the range of impact velocities assessed for the V_50_ evaluations. The slope and the intercept of the linear fittings reflect the performance of the material in terms of protection for the soft-tissue simulant behind it. In this consideration, the best performing material is again Dyneema^®^ HPPE knit, followed by Kevlar^®^ felt. The least performing material is the dilatant fabric, followed by Twaron^®^ weave 1. The other three fabrics show a mixed performance. Compared with tests on gelatine only with no protective fabric from the study by Nguyen et al. (2018) with the same FSP (dotted line in Figure 3), all tested fabrics resulted in smaller DoP, meaning less damage to the soft-tissue simulant, for the same impact velocity.

The normalized percentage energy absorption values of tested materials [category (e)] between 100 to 200 m/s impact velocity are listed in Table 2. According to the statistical *t*-test and the Bonferroni multiple-comparison correction (α_*corrected*_ = 0.05/21≈0.002), there were significant differences in the values between all the fabrics apart from the pairs Kevlar^®^ knit – Kevlar^®^ felt and Kevlar^®^ felt – dilatant fabric (Supplementary Table SE). In terms of the normalized energy absorption, the fabrics can be grouped as Group 1: Kevlar^®^ weave and Twaron^®^ weave 1 – the most effective in energy absorption per unit of mass – followed by Group 2: Twaron^®^ weave 2; Group 3: Kevlar^®^ knit, Kevlar^®^ felt; Group 4: Kevlar^®^ felt, dilatant fabric, and lastly Group 5: Dyneema^®^ HPPE knit.

**TABLE 2:** The normalized percentage energy absorption of each investigated fabric in single-layer format [category (e)] calculated for the range of impact velocity between 100 and 200 m/s.

**Material**	**$\hat{E}$ [10^–3^ %/gsm]**	**Group**
Kevlar^®^ plain weave	8.00 ± 1.11	1
Twaron^®^ plain weave 1	8.00 ± 0.80	1
Twaron^®^ plain weave 2	5.26 ± 0.47	2
Kevlar^®^ felt	3.64 ± 0.67	3
Dilatant fabric	3.33 ± 0.21	3, 4
Kevlar^®^ knit	3.85 ± 0.95	4
Dyneema^®^ HPPE knit	1.64 ± 0.66	5

### Effect of Ballistic Gelatine Backing and V_50_ Evaluation Methods on Outcomes

**FIGURE 5:**
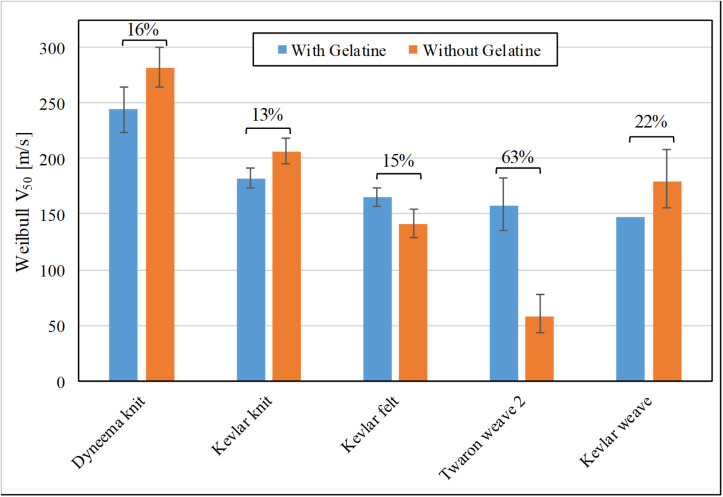
The FSP impact velocity with 50% risk of material perforation for experiments with (blue) and without (orange) ballistic gelatine backing; the percentage difference relative to the earlier is labeled for each single-layer material.

The V_50_ values of material perforation by the FSP for the two assessed boundary conditions (with and without soft-tissue simulant as backing material) for the five ballistic materials tested are shown in Figure 5. The percentage difference – the difference between the two V_50_ values relative to the V_50_ value with ballistic gelatine – for each fabric is also displayed. Kevlar^®^ knit shows the smallest discrepancy (13%), whereas Twaron^®^ weave 2 shows the greatest discrepancy (63%).

**FIGURE 6:**
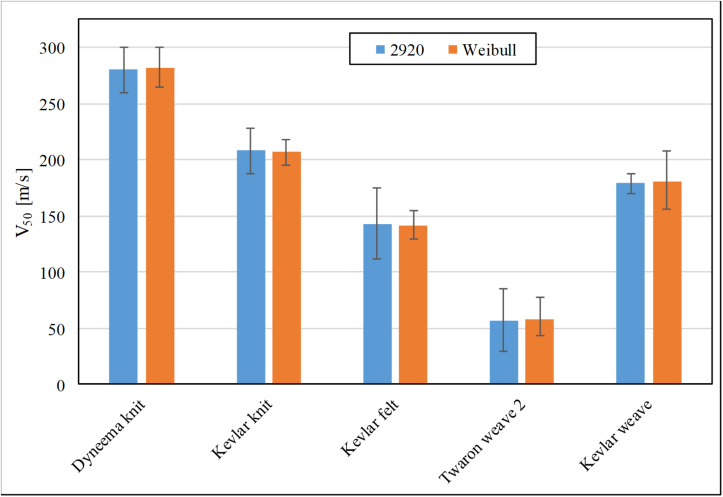
The FSP impact velocity with 50% risk of material perforation by FSP calculated using the AEP-2920 NATO Standard method (blue) and the Weibull survivability analysis (orange).

Figure 6 compares the V_50_ values for the five studied materials, without the ballistic gelatine backing, obtained by the two evaluation methods. The error bar for the method proposed by the AEP-2920 NATO Standard is the corresponding velocity window of the six impact velocities used for the calculation. The error bar for the Weibull analysis is either the 95% confidence interval or half-width of the impact velocity gap, as described earlier for results with soft-tissue simulant. For all cases, the percentage difference of each pair of V_50_ values is less than 2%.

## Discussion

All investigated single-layer ballistic materials were shown to reduce the damage to the soft-tissue simulant in terms of DoP of the FSP. Among them, Dyneema^®^ HPPE knit demonstrated the best performance when weight is not considered, but the worst performance in terms of energy attenuation per unit mass. This suggests that the protection benefits provided by Dyneema^®^ knit over other materials are mainly thanks to its outstanding high density. In addition, there is an overall trend that the higher the areal density, the better the ballistic fabric can reduce DoP in gelatine [category (d)], in other words result in shallower penetration. This is expected as it has been shown that the areal density directly affects the ballistic limit and energy absorption capacity of fabrics, and thus the trauma resulting from the energy that cannot be absorbed by them (Billon and Robinson, 2001; Jacobs and Van Dingenen, 2001; Karahan et al., 2008; Breeze et al., 2013a). The exception was the dilatant fabric, which demonstrated the lowest performance across assessed categories (a) to (d) despite having the second highest areal density. This is likely due to the lamination fixing the yarns in place and reducing the inter-yarn friction, hence, limiting the energy dissipation throughout the woven material (Karahan et al., 2008). In the case of Twaron^®^ plain weave 1 and 2 fabrics which are the same yarn material but of different areal densities, the denser fabric expectedly shows better performance across categories [(a) to (d)], but with lower normalized percentage energy absorption [category (e)]. It was observed that the denser Twaron^®^ weave has a larger yarn, or lower sett (yarns/10 mm), which is likely the reason for the lower protection effectiveness (Karahan et al., 2008; Sakaguchi et al., 2012). These two observations prove that density is not the only deciding factor toward the performance of the materials, but their architectures are of equal, if not greater, importance (Karahan et al., 2008; Petrulis, 2012; Tran et al., 2014; Wang et al., 2014).

Knitted materials (Kevlar^®^ knit and Dyneema^®^ HPPE knit) are generally more resistant against perforation than protective for the soft-tissue simulant behind. Due to their elasticity, most knitted fabrics can exhibit large deformations, allowing them to be resistant against perforation by the FSP. However, it also allows for the FSP to travel forward more easily into the soft tissue simulant (penciling). This is in line with the numerical study by Tran et al. (2014) showing that a knitted fabric gives lower ballistic performance than a plain-woven fabric of similar areal density. This means that the knitted fabric is more effective in stopping ingress of foreign particles such as dirt, small fragments, or soil ejecta into the body than mitigating injuries to the soft tissue.

The two evaluation methods of the V_50_ impact velocity were in good agreement with each other indicating that either method is suitable for a small number of impact tests. For larger amounts of data, the Weibull probability distribution is likely to provide a more accurate estimation of V_50_ compared to the arithmetic mean estimation proposed by the AEP-2920 NATO Standard. As the set of data used for AEP-2920 method falls within the 40–60 m/s bracket, it means that the error can be up to 20–30 m/s, even for an increased amount of data points. In this case, the 95% confidence interval obtained from the survivability analysis should be a more precise and meaningful indication of error in the evaluation.

Ballistic gelatine as soft-tissue simulant was chosen to be the backing material in the primary experiment to obtain a biofidelic boundary condition behind the tested fabric. It is most relevant for body areas that are covered by large amount of muscle, such as the thigh or neck. It is likely representative for the abdomen, although this body area has several air-tissue interfaces. Our results show that lack of a soft-tissue simulant backing will give rise to inaccurate and unpredictable changes in the behavior of the ballistic materials since the biofidelic interaction between the fabric and the soft tissue is missing. The importance of correct boundary condition shown here also suggests that using other, less biofidelic backing materials, such as AlCuMg-alloy witness sheets or Plasticine clay boards (Sakaguchi et al., 2012; NATO, 2016) may not provide realistic responses of the investigated materials. Furthermore, the ballistic gelatine can quantitatively reflect the damage inflicted on the soft tissues through DoP measurements, bringing the focus of the protection assessment to the injury behind the amour. It also enables the estimation of the energy absorbed by the fabric, which is a useful metric for comparing different materials. With these advantages, the inclusion of a biofidelic backing material such as ballistic gelatine would be a meaningful and beneficial addition to the current testing standards for ballistic fabrics (NATO, 2016).

The reported results are specific for the FSP used. Instead of the traditional 1.10-g chisel-nosed FSP, the 0.78-g cylindrical FSP chosen was based on evidence that it is more representative of fragments causing injury in recent conflicts (Breeze et al., 2013d) and available literature studying the risk of blast fragment penetrating injury (Nguyen and Masouros, 2019; Nguyen et al., 2020) which are useful to assess the protection in terms of improved injury outcomes. A more comprehensive understanding of the responses of these ballistic materials to fragment impacts can be achieved by repeating the experimental model with other FSPs such as ball bearings, chisel-nosed cylinders, and soil ejecta. Due to differing geometries and compositions, these FSPs are likely to exhibit quite different interactions with protective fabrics (Bazhenov, 1997; Cheeseman and Bogetti, 2003; Tan et al., 2003; Tan and Khoo, 2005; Li et al., 2016). Blunt projectiles like ball bearings do not have angled edges and cannot easily slip between the yarns so they are subjected to more deceleration by the fabric (Bazhenov, 1997; Lim et al., 2002; Tan et al., 2003; Manes et al., 2014). Flat-head projectiles with sharp edges, such as the one used in this study, shear through the yarns of the fabric, resulting in a lower attenuation of their energy compared to blunt projectiles (Prosser et al., 2000; Lim et al., 2002; Tan et al., 2003). Projectiles with pointed head or narrow nose such as the chisel-nosed cylinder can wedge through the fabric and thus can perforate it at lower impact velocities compared to other shapes (Prosser et al., 2000; Lim et al., 2002; Tan et al., 2003; Manes et al., 2014). Particulates from soil ejecta with small size like sand grains can infiltrate between the fabric fibers and infect the soft tissue behind (Saunders and Carr, 2018). These interactions also depend on the mass and diameter of the FSPs (Cheeseman and Bogetti, 2003; Naik et al., 2005), so additional experiments need to be carried out to quantify the effect of these parameters on the protective ability of the chosen fabrics. To expand these results, testing needs to be performed using multiple layers of the materials tested in this study. Each layer will increase the V_50_ required for penetration, although previous research would suggest that the correlation is not linear and also dependant on the geometry of the projectile (Lim et al., 2002; Sakaguchi et al., 2012).

When used in practice, these fabrics are likely to be added onto existing clothing; thus, testing them together with the intended clothing fabric will give even more relevant boundary conditions. In addition, the ballistic gelatine was used to represent both muscle and skin; whilst gelatine is a good surrogate for muscle tissue, it is less resistant to FSP penetration than skin at lower velocities (Breeze and Clasper, 2013). Furthermore, the well-controlled experiments allowed for a reproducible performance and therefore fair comparisons between fabrics and whether a backing material affects assessments. Environmental factors such as temperature, humidity, impurity, and dampness, to name but a few that would vary depending on the location of the field, would all contribute to the performance of the protective fabrics. Immersion in water (Bazhenov, 1997; Li et al., 2016) and domestic laundering (Helliker et al., 2014) have been shown to reduce the performance of some ballistic protective fabrics. The effect of these factors on the protective ability of protective fabrics should be determined experimentally, and the proposed apparatus and methodology presented here offers a reproducible test bed to do so.

## Conclusion

This study compared the ballistic performance of various commercially available fabrics and quantified the effect of including a biofidelic backing material on assessing ballistic protection. Inclusion of a biofidelic material was shown to have a marked effect on the ballistic protective qualities of the fabrics from FSPs. Therefore, adopting the inclusion of a biofidelic backing material in qualification tests of soft ballistic protection, at least against small fragments, is highly recommended.

It was shown that even a single layer of ballistic fabric could provide meaningful protection. Further testing on the hybrid of two layers from different fabrics is recommended to utilize optimally the combined performance of these fabrics. Testing with the presence of the existing clothing would improve the relevance of the testing method.

## Data Availability Statement

The raw data supporting the conclusions of this article will be made available by the authors, without undue reservation.

## Author Contributions

SM and JB conceived the project idea which was developed into an experimental model by T-TN and GM. All the tests, including preparation of samples and data acquisition, were carried out by T-TN and GM. The data analysis was conducted by T-TN and GM. T-TN drafted the manuscript. All authors contributed to the article and approved the submitted version.

## Conflict of Interest

The authors declare that the research was conducted in the absence of any commercial or financial relationships that could be construed as a potential conflict of interest.
