# Supplementary material for: Gelatine Backing Affects the Performance of Single-Layer Ballistic-Resistant Materials Against Blast Fragments
Source: Front Bioeng Biotechnol. 2020 Jul 2;8:744. doi: 10.3389/fbioe.2020.00744 (PMC7343711; doi:10.3389/fbioe.2020.00744)
Supplement: Supplementary file 1 [file Table_1.DOCX]

Supplementary Material

| Table A – Statistic z-test and Bonferroni multiple-comparison correction ($\propto=0.002$) for V_50_ of material perforation | | | | | | | |
| --- | --- | --- | --- | --- | --- | --- | --- |
| p-value  z-score | Kevlar weave | Twaron weave 1 | Twaron weave 2 | Kevlar knit | Kevlar  felt | Dilatant fabric | Dyneema knit |
| Kevlar weave |  | < 10^-5^ | 0.45 | < 10^-5^ | 0.56 × 10^-4^ | NA | < 10^-5^ |
| Twaron weave 1 | 11.92 |  | 0.04 | < 10^-5^ | < 10^-5^ | NA | < 10^-5^ |
| Twaron weave 2 | 0.752 | 2.03 |  | 0.05 | 0.51 | NA | < 10^-5^ |
| Kevlar knit | 7.942 | 11.06 | 2.00 |  | 0.01 | NA | < 10^-5^ |
| Kevlar felt | 4.03 | 7.30 | 0.67 | 2.77 |  | NA | < 10^-5^ |
| Dilatant fabric | NA | NA | NA | NA | NA |  | NA |
| Dyneema knit | 9.41 | 10.84 | 5.53 | 5.55 | 7.08 | NA |  |

| Table B – Statistic z-test and Bonferroni multiple-comparison correction ($\propto=0.002$) for V_50_ of gelatine penetration | | | | | | | |
| --- | --- | --- | --- | --- | --- | --- | --- |
| p-value  z-score | Kevlar weave | Twaron weave 1 | Twaron weave 2 | Kevlar knit | Kevlar  felt | Dilatant fabric | Dyneema knit |
| Kevlar weave |  | < 10^-5^ | 0.0071 | < 10^-5^ | 0.58 | NA | < 10^-5^ |
| Twaron weave 1 | 5.70 |  | 0.23 | 0.076 | 0.040 | NA | < 10^-5^ |
| Twaron weave 2 | 2.69 | 1.20 |  | 0.65 | 0.019 | NA | < 10^-5^ |
| Kevlar knit | 5.30 | 1.77 | 0.45 |  | 0.0081 | NA | < 10^-5^ |
| Kevlar felt | 0.56 | 2.06 | 2.34 | 2.65 |  | NA | 5.57 × 10^-4^ |
| Dilatant fabric | NA | NA | NA | NA | NA |  | NA |
| Dyneema knit | 8.096 | 11.03 | 6.40 | 10.55 | 3.45 | NA |  |

| Table C – Statistic z-test and Bonferroni multiple-comparison correction ($\propto=0.002$) for V_50_ of gelatine penetration > 15 mm | | | | | | | |
| --- | --- | --- | --- | --- | --- | --- | --- |
| p-value  z-score | Kevlar weave | Twaron weave 1 | Twaron weave 2 | Kevlar knit | Kevlar  felt | Dilatant fabric | Dyneema knit |
| Kevlar weave |  | 0.051 | 0.45 | < 10^-5^ | < 10^-5^ | 0.31 | < 10^-5^ |
| Twaron weave 1 | 1.95 |  | 0.21 | < 10^-5^ | < 10^-5^ | 0.65 | < 10^-5^ |
| Twaron weave 2 | 0.75 | 1.25 |  | 0.31 | 0.16 | 0.21 | < 10^-5^ |
| Kevlar knit | 9.31 | 6.94 | 1.02 |  | 0.056 | 0.0051 | < 10^-5^ |
| Kevlar felt | 45.58 | 9.69 | 1.42 | 1.91 |  | 0.0012 | < 10^-5^ |
| Dilatant fabric | 1.02 | 0.45 | 1.25 | 2.80 | 3.24 |  | < 10^-5^ |
| Dyneema knit | 9.41 | 9.55 | 5.53 | 7.12 | 6.85 | 6.94 | - |

| Table D – The statistical difference in overall performance of all investigated fabrics | | | | | | | |
| --- | --- | --- | --- | --- | --- | --- | --- |
|  | Kevlar weave | Twaron weave 1 | Twaron weave 2 | Kevlar knit | Kevlar  felt | Dilatant fabric | Dyneema knit |
| Kevlar weave |  | 2 | 0 | 3 | 2 | NA | 3 |
| Twaron weave 1 |  |  | 0 | 2 | 2 | NA | 3 |
| Twaron weave 2 |  |  |  | 0 | 0 | NA | 3 |
| Kevlar knit |  |  |  |  | 0 | NA | 3 |
| Kevlar felt |  |  |  |  |  | NA | 3 |
| Dilatant fabric |  |  |  |  |  |  | NA |
| Dyneema knit |  |  |  |  |  |  |  |

| Table E – Statistic t-test and Bonferroni multiple-comparison correction ($\propto=0.002$) for V_50_ of normalised energy absorption | | | | | | | |
| --- | --- | --- | --- | --- | --- | --- | --- |
| p-value  t-score | Kevlar weave | Twaron weave 1 | Twaron weave 2 | Kevlar knit | Kevlar  felt | Dilatant fabric | Dyneema knit |
| Kevlar weave |  | 0.86 | < 10^-5^ | < 10^-5^ | < 10^-5^ | < 10^-5^ | < 10^-5^ |
| Twaron weave 1 | -0.18 |  | < 10^-5^ | < 10^-5^ | < 10^-5^ | < 10^-5^ | < 10^-5^ |
| Twaron weave 2 | 6.37 | 8.36 |  | < 10^-5^ | < 10^-5^ | < 10^-5^ | < 10^-5^ |
| Kevlar knit | 12.34 | 16.80 | 9.81 |  | 0.12 | 0.00014 | < 10^-5^ |
| Kevlar felt | 11.11 | 15.35 | 9.90 | 1.59 |  | 0.0041 | < 10^-5^ |
| Dilatant fabric | 10.86 | 14.44 | 9.79 | 4.59 | 3.29 |  | < 10^-5^ |
| Dyneema knit | 12.37 | 17.30 | 17.51 | 19.56 | 21.38 | 8.081 |  |
